# Supplementary material for: Spermine modulation of Alzheimer’s Tau and Parkinson’s α-synuclein: implications for biomolecular condensation and neurodegeneration
Source: Nat Commun. 2025 Nov 21;16:10239. doi: 10.1038/s41467-025-65426-3 (PMC12639133; doi:10.1038/s41467-025-65426-3)
Supplement: Supplementary file 1 — Supplementary Information [file 41467_2025_65426_MOESM1_ESM.pdf]

Supporting Information

Spermine Modulation of Alzheimer's Tau and Parkinson's α-Synuclein: Implications for Biomolecular Condensation and Neurodegeneration

Xun Sun[1], Debasis Saha[2], Xue Wang[1], Cecilia Mörman[1, 3], Rebecca Sternke-Hoffmann[1], Juan Atilio Gerez[4], Fátima Herranz-Trillo[5], Roland Riek[4], Wenwei Zheng[2], Jinghui Luo[1,*]

[1.] Center for Life Sciences, Paul Scherrer Institute, Forschungsstrasse 111, 5232 Villigen PSI, Switzerland
[2.] College of Integrative Sciences and Arts, Arizona State University, Mesa, AZ, 85212, United States
[3.] Department of Medicine Huddinge, Division of Biosciences and Nutrition, Karolinska Institutet, 14152 Huddinge, Sweden
[4.] Institute of Molecular Physical Science, Department of Chemistry and Applied Biosciences, ETH Zurich, Zurich, Switzerland
[5.] CoSAXS Beamline, MAX IV Laboratory, Lund, Sweden
* Corresponding authors. E-mail address: jinghui.luo@psi.ch

Supporting Methods

Primary sequence of proteins in this study

Tau

MAEPRQEFEVMEDHAGTYGLGDRKDQGGYTMHQDQEGDTDAGLKESPLQTPTEDGSEEPGSETS
DAKSTPTAEDVTAPLVDEGAPGKQAAAQPHTEIPEGTTAEEAGIGDTPSLEDEAAGHVTQARMVSKS
KDGTGSDDKKAKGADGKTKIATPRGAAPPGQKGQANATRIPAKTPPAPKTPPSSGEPPKSGDRSGYS
SPGSPGTPGSRSRTPSLPTPPTREPKKVAVVRTPPKSPSSAKSRLQTAPVPMPDLKNVKSKIGSTENL
KHQPGGGKVQIINKKLDLSNVQSKCGSKDNIKHVPGGGSVQIVYKPVDLSKVTSKCGSLGNIHHKPG
GGQVEVKSEKLDFKDRVQSKIGSLDNITHVPGGGNKKIETHKLTFRENAKAKTDHGAEIVYKSPVVSG
DTSPRHLSNVSSTGSIDMVDSPQLATLADEVSASLAKQGL

K18

QTAPVPMPDLKNVKSKIGSTENLKHQPGGGKVQIINKKLDLSNVQSKCGSKDNIKHVPGGGSVQIVYK
PVDLSKVTSKCGSLGNIHHKPGGGQVEVKSEKLDFKDRV QSKIGSLDNITHVPGGGNKKIE

αS

MDVFMKGLSKAKEGVVAAAEKTKQGVAEAAGKTKEGVLYVGSKTKEGVVHGVATVAEKTKEQVTNV
GGAVVTGVTAVAQKTVEGAGSIAAATGFVKKDQLGKNEEGAPQEGILEDMPVDPDNEAYEMPSEEGY
QDYEPEA

Supporting Figures

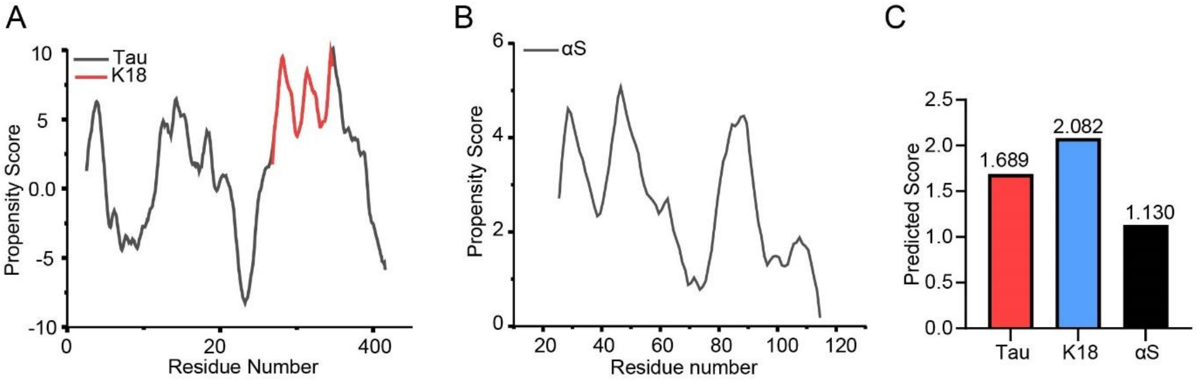

Supplementary Figure. 1: Propensity Score prediction of Tau, K18 and αS. A-B, Residue-specific propensity score for LLPS predicted by catGranule for Tau, K18 (**A**) and αS (**B**). **C,** Total catGranule-scores of Tau, K18 and αS.

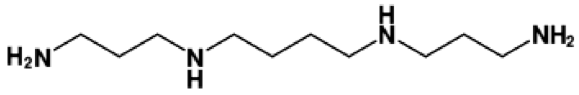

Supplementary Figure. 2: the chemical structure of spermine

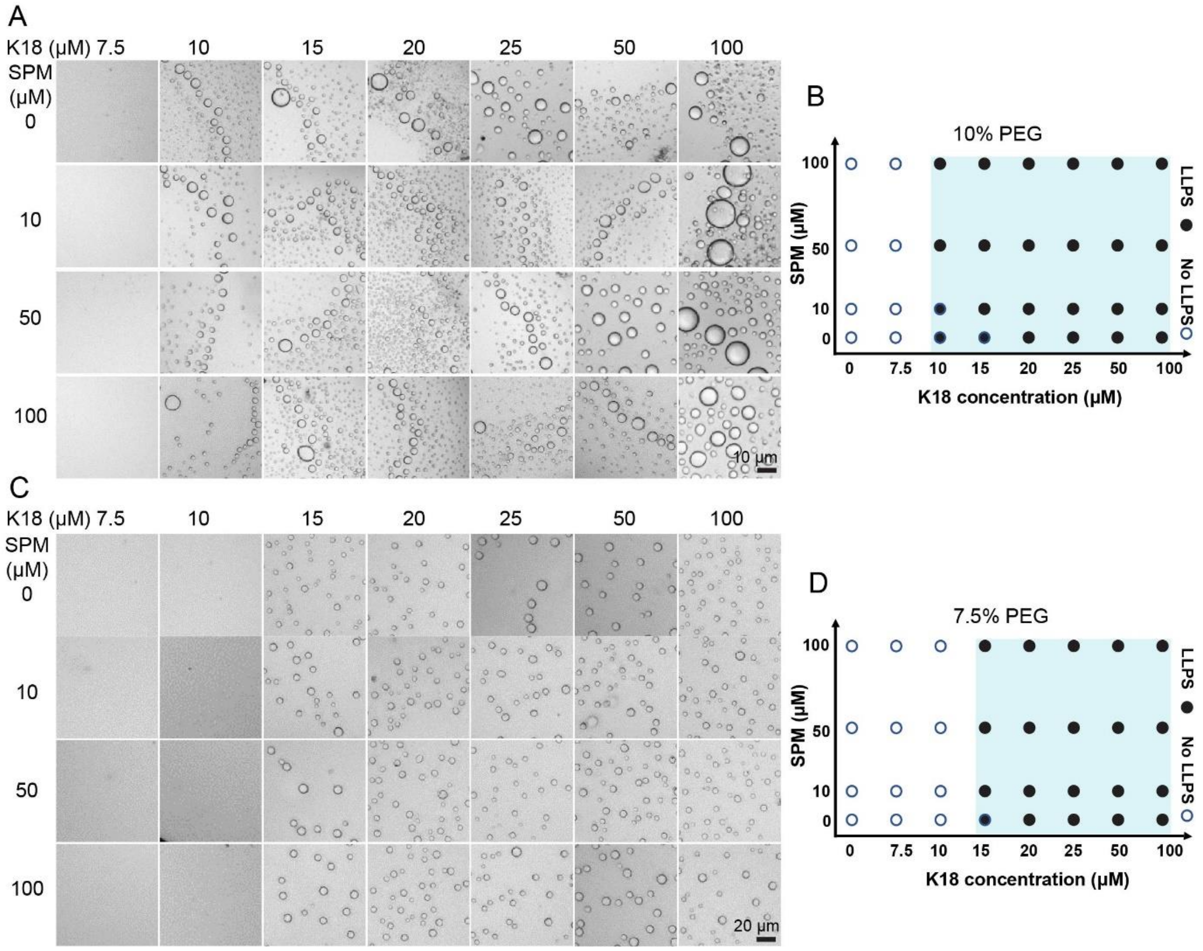

Supplementary Figure. 3: Spermine does not change the propensity of K18 LLPS *in vitro*. A-B, Representative images (**A**) and corresponding phase diagram (**B**) of phase separated droplets in the presence of various concentrations of K18 and spermine showing the regime of K18 LLPS in the presence of 10% PEG. Scale bar in the images is 10 μm. **C-D**, Representative images (**C**) and corresponding phase diagram (**D**) of phase separated droplets in the presence of various concentrations of K18 and spermine showing the regime of K18 LLPS in the presence of 7.5% PEG. Scale bar in the images is 20 μm. n=3.

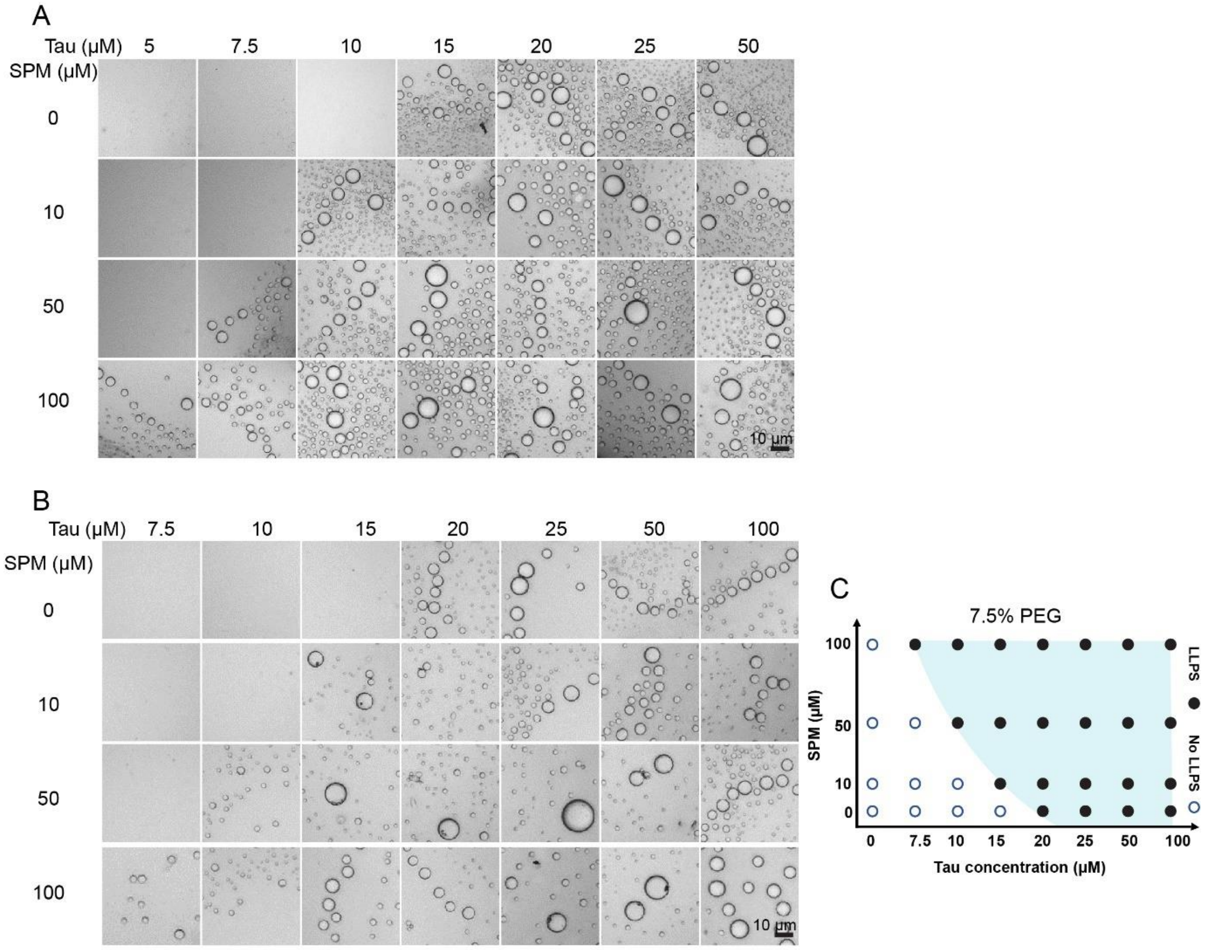

Supplementary Figure. 4: Spermine increases the propensity of Tau LLPS *in vitro*. A, Representative images of phase separated droplets in the presence of various concentrations of Tau and spermine showing the regime of Tau LLPS in the presence of 10% PEG. Scale bar in the images is 10 μm. **B-C**, Representative images (**B**) and corresponding phase diagram (**C**) of phase separated droplets in the presence of various concentrations of Tau and spermine showing the regime of Tau LLPS in the presence of 7.5% PEG. Scale bar in the images is 10 μm. n=3

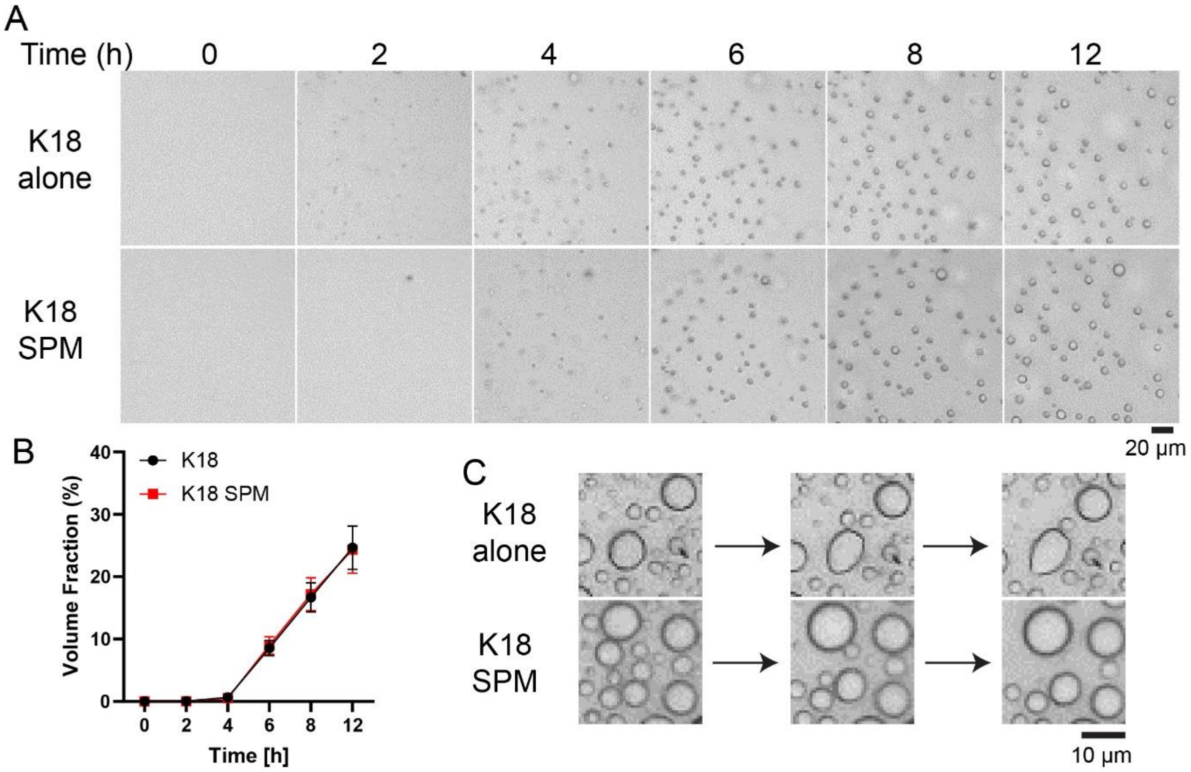

Supplementary Figure. 5: Spermine does not change the property of K18 LLPS *in vitro*. A, Representative time lapse images of phase separated droplets in the 10 µM of K18 in the absence and presence of 100 µM spermine in the presence of 10% PEG. Scale bar in the images is 20 µm. **B**, Quantification of the condensates volume fraction (n=3). **C**, liquid-like droplet fusion of 10 µM of K18 in the absence and presence of 100 µM spermine in the presence of 10% PEG. Scale bar in the images is 10 µm. n=3

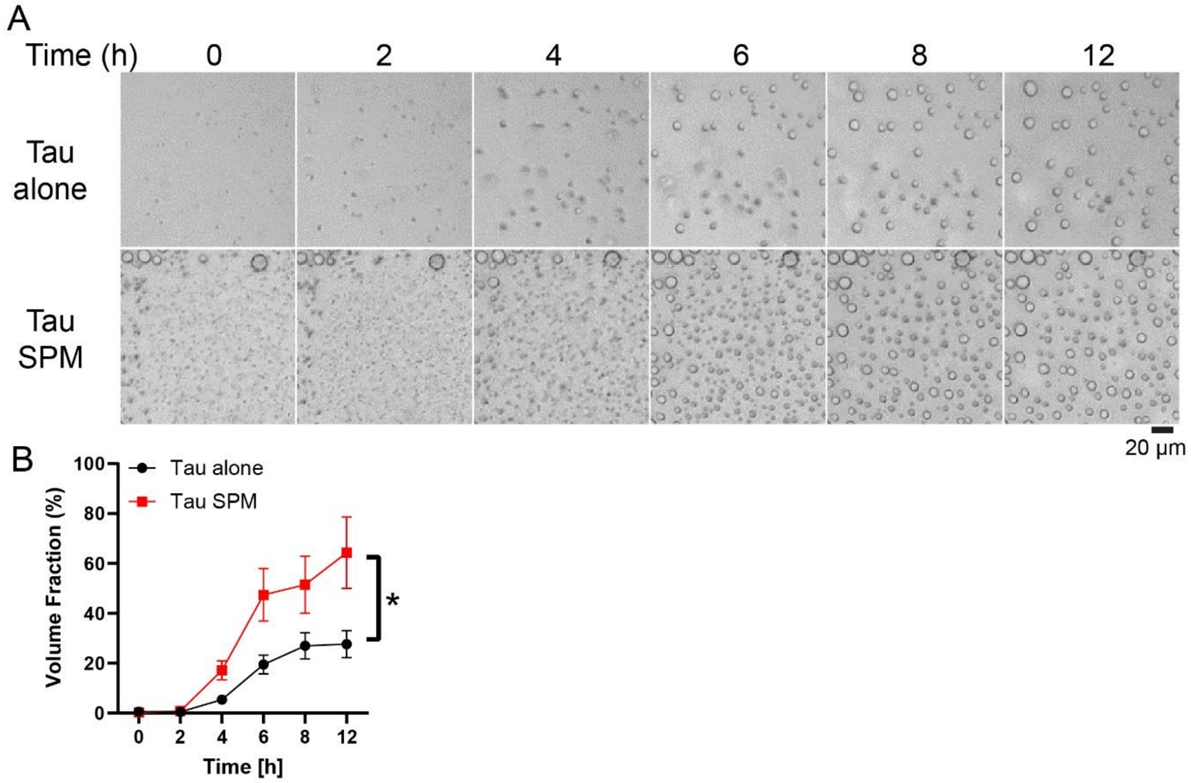

Supplementary Figure. 6: Spermine accelerates Tau LLPS *in vitro*. A, Representative time lapse images of phase separated droplets in the 20 μM of Tau in the absence and presence of 100 μM spermine in the presence of 10% PEG. Scale bar in the images is 20 μm. **B**, Quantification of the condensates volume fraction (n=3) corresponding to A.

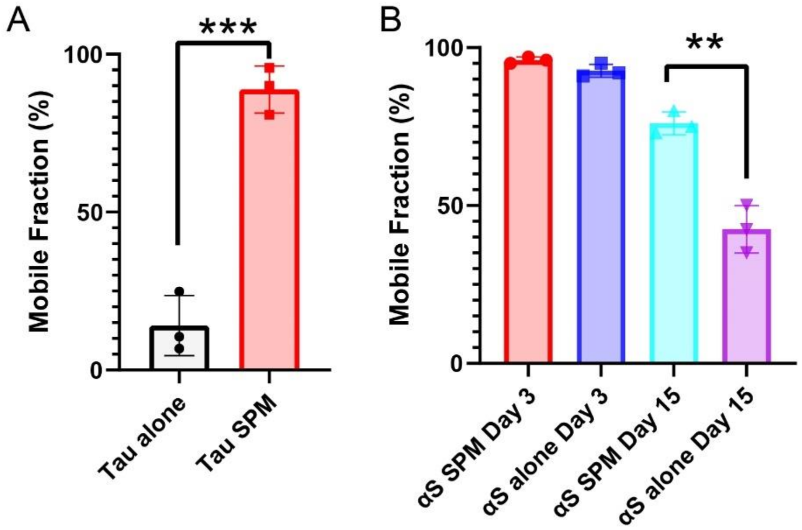

Supplementary Figure. 7: Quantification of the mobile fraction from FRAP experiments for Tau with or without SPM (A) corresponding to Figure 1D and αS with or without SPM (B) corresponding to Figure 1J. n=3.

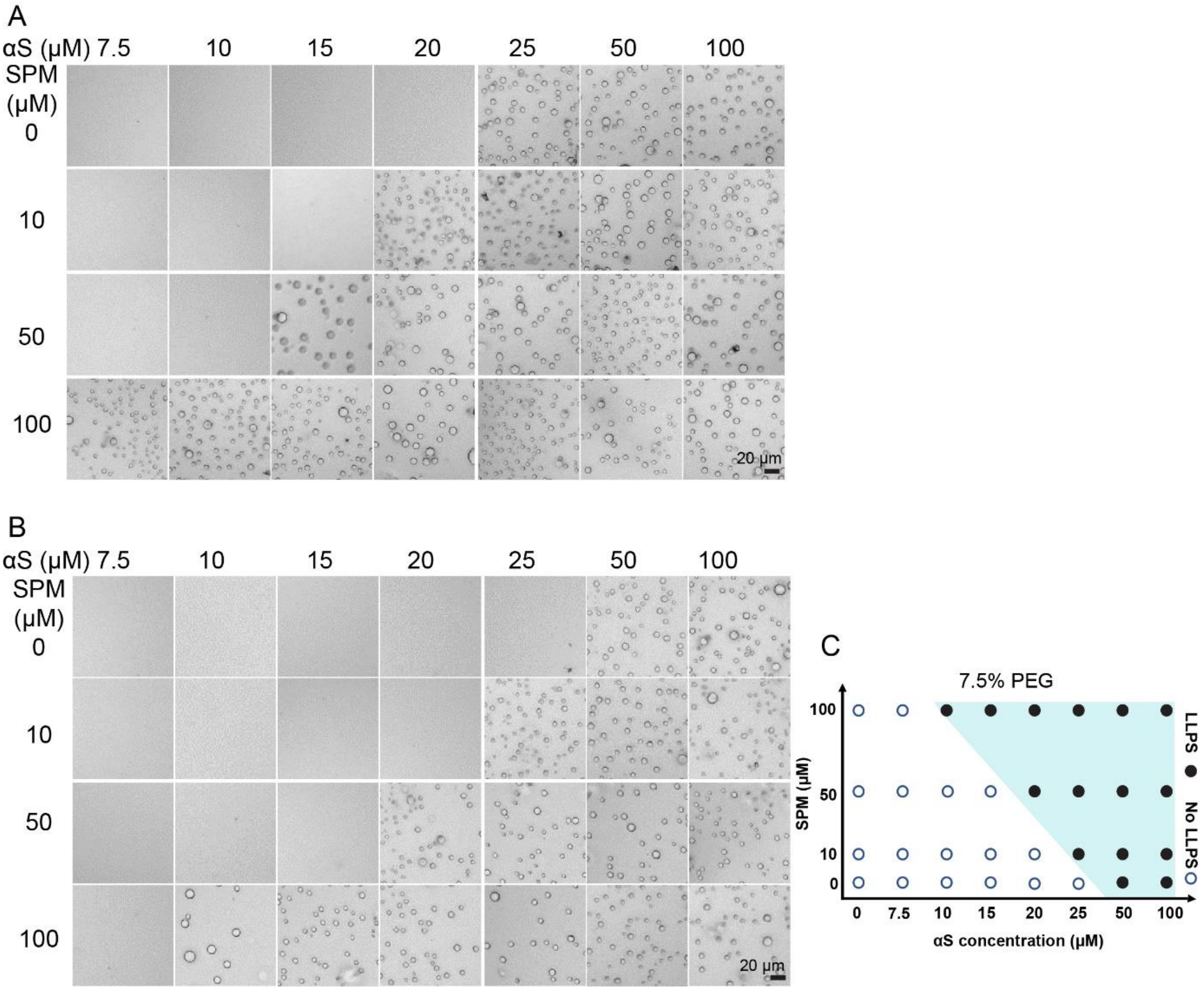

Supplementary Figure. 8: Spermine increases the propensity of αS LLPS *in vitro*. A, Representative images of phase separated droplets in the presence of various concentrations of αS and spermine showing the regime of αS LLPS in the presence of 10% PEG. Scale bar in the images is 20 μm. **B-C**, Representative images (**B**) and corresponding phase diagram (**C**) of phase separated droplets in the presence of various concentrations of αS and spermine showing the regime of αS LLPS in the presence of 7.5% PEG. Scale bar in the images is 20 μm. n=3,

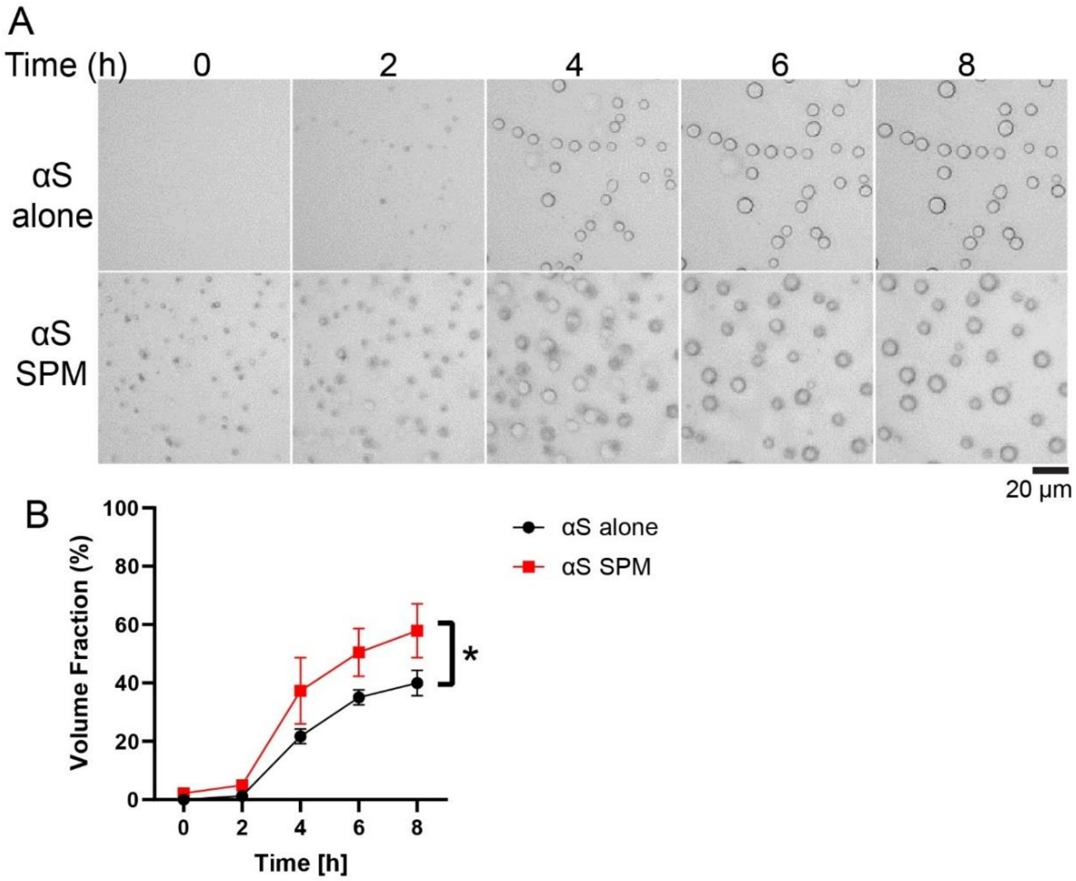

Supplementary Figure. 9: Spermine accelerates αS LLPS *in vitro*. A, Representative time lapse images of phase separated droplets in the 25 µM of αS in the absence and presence of 100 µM spermine in the presence of 10% PEG. Scale bar in the images is 20 µm. **B**, Quantification of the condensates volume fraction (n=3) corresponding to A. n=3.

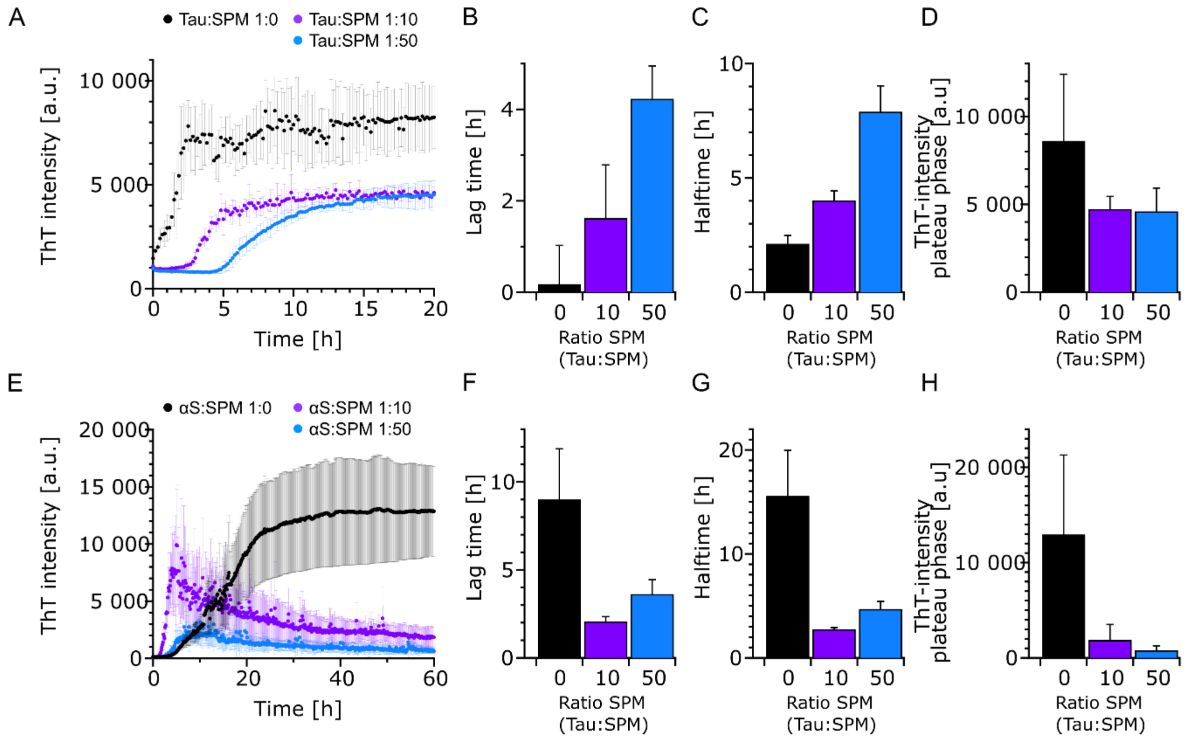

Supplementary Figure. 10: Spermine suppresses amyloid fibrilliation of Tau and αS in the condensate state. A, Thioflavin T (ThT) fluorescence assay monitoring Tau fibril formation over time in the absence and presence of SPM at molar ratios of 1:10 and 1:50 (Tau:SPM). (**B-D**) Quantification of kinetic parameters from (**A**), including lag time (**B**), half-time (**C**), and ThT plateau intensity (**D**). (**E**) ThT kinetics of αS aggregation with or without SPM at the indicated ratios. (**F-H**) Quantification of αS aggregation kinetics showing lag time (**F**), half-time (**G**), and plateau intensity (**H**). Error bars represent standard deviations from five replicates. Buffer: 25 mM HEPES, pH 7.4, 10% PEG8000, 20 μM ThT.

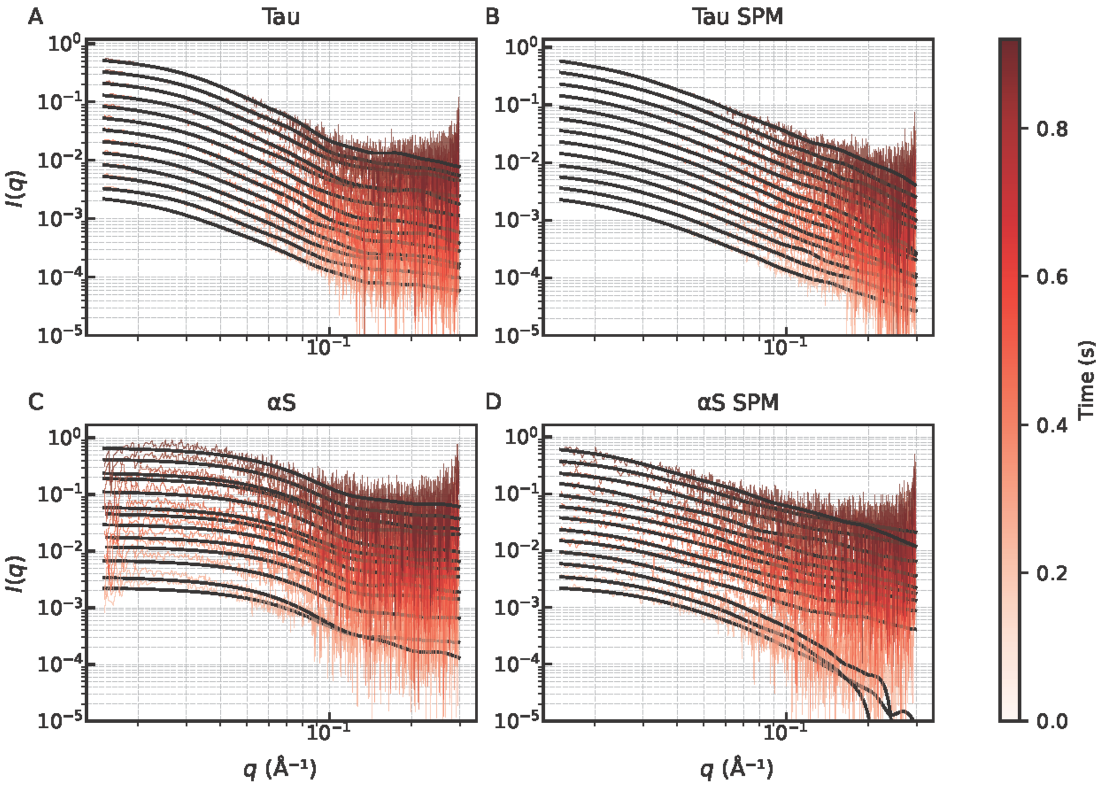

Supplementary Figure. 11: TR-SAXS measurement and EOM fitting of Tau and αS in the absence and presence of spermine. A-B, Experimental TR-SAXS data points and EOM fitting (black) plots of Tau in the absence (A) and presence of spermine (B) are displayed. Experimental curves from early time points are shown red, to late time points in black, with a color gradient to indicate time evolution for in-between time points. **C-D**, Experimental TR-SAXS data points and EOM fitting (black) plots of αS in the absence (C) and presence of spermine (D) are displayed.

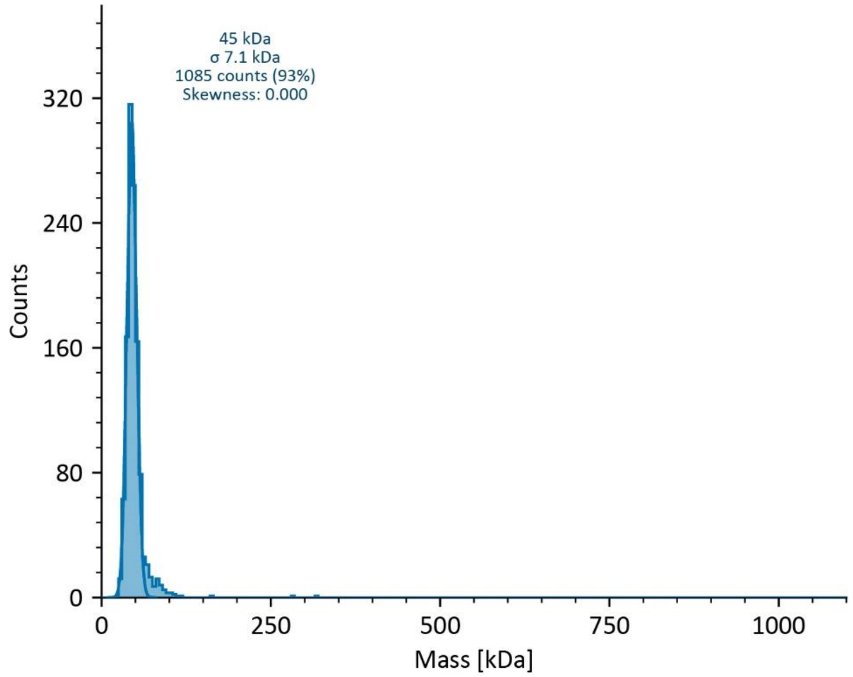

Supplementary Figure. 12: Mass photometry measurement of Tau sample shows a predominant presence of monomeric Tau. Tau protein was prepared in 25 mM HEPES buffer (pH 7.4) and maintained on ice. A 2 µL of the sample was added to adjusted to a final concentration of 10 nM, sample was measured within 30 minutes of preparation.

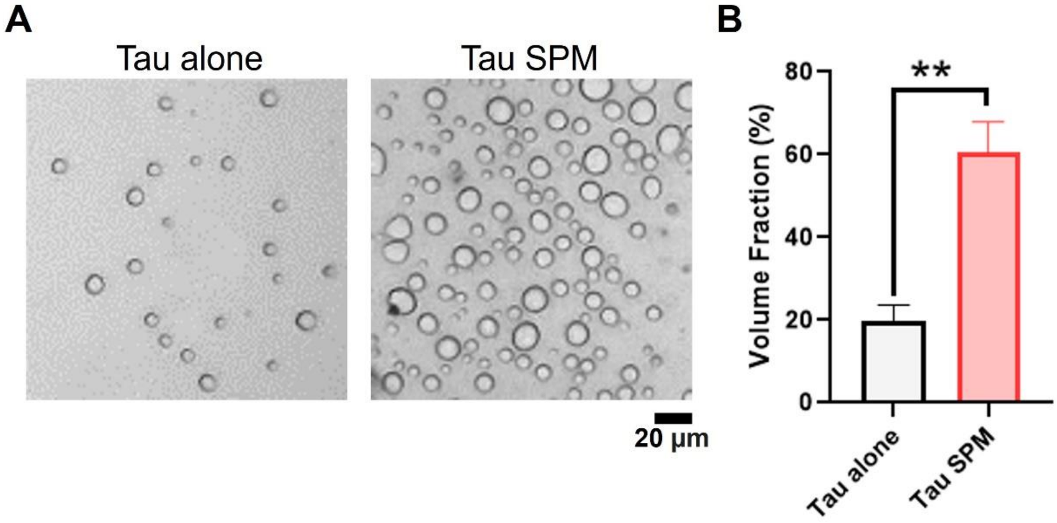

Supplementary Figure. 13: Spermine increases the propensity of Tau LLPS in phosphate buffer (20 mM Na_2HPO_4/NaH_2PO_4, pH 6.3). A, Representative images of phase separated 20 µM Tau droplets in the absence and presence of 100 µM spermine. **B,** Quantification of droplet volume fraction showing that spermine increases Tau LLPS in phosphate buffer used for NMR measurement. Buffer condition: 20 mM Na_2HPO_4/NaH_2PO_4, pH 6.3, 10% PEG8000. n=3

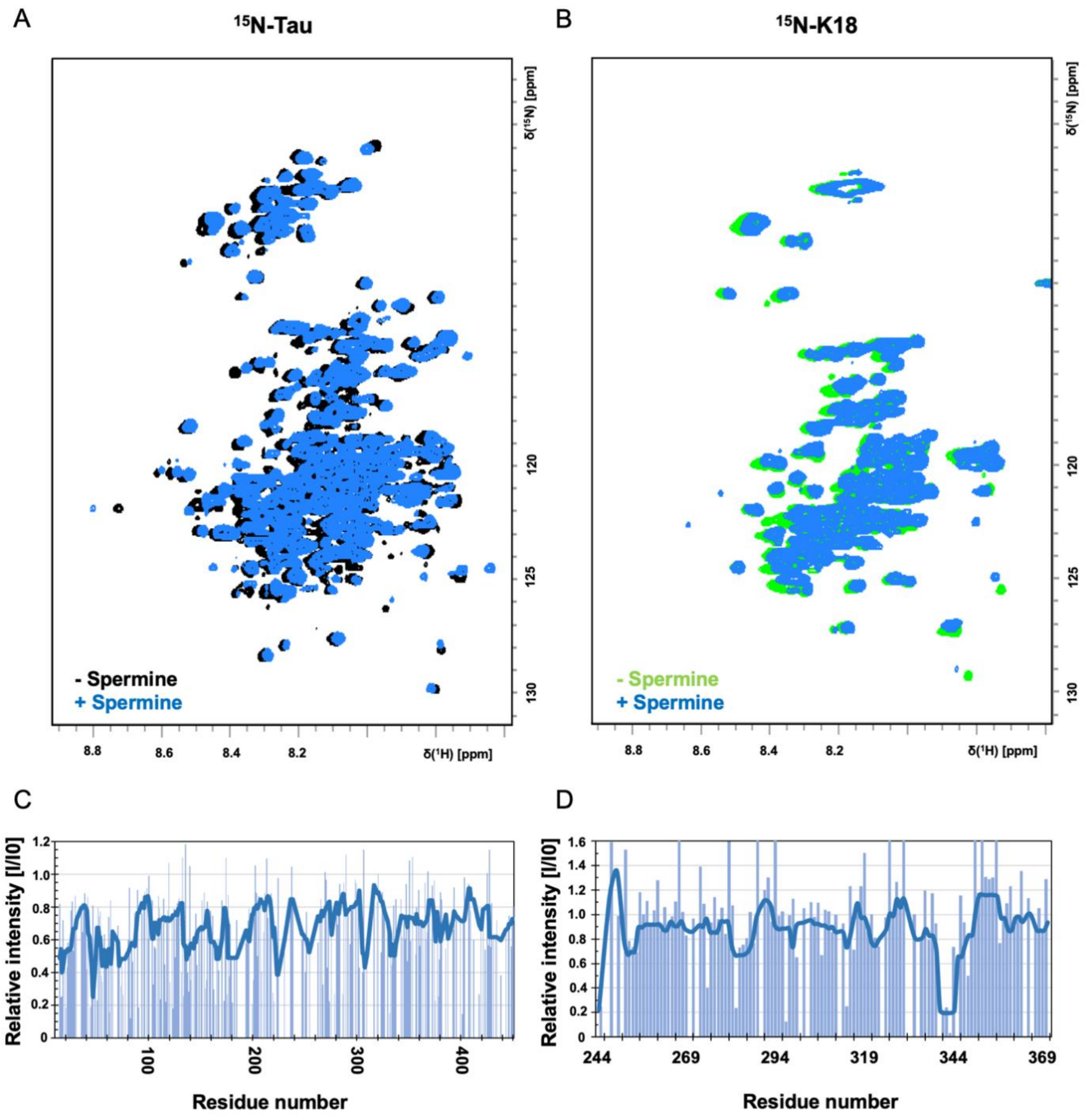

Supplementary Figure. 14: 2D ^{1}H-^{15}N-HMQC NMR measurements an interaction between spermine and full-length Tau-441 protein and K18. Full-sized spectra of full-length ^{15}N-Tau (Tau-441) (A) and ^{15}N-K18 (B) in the absence and presence of spermine in PBS buffer with a pH around 6.3. The spectra were recorded at 283 K.

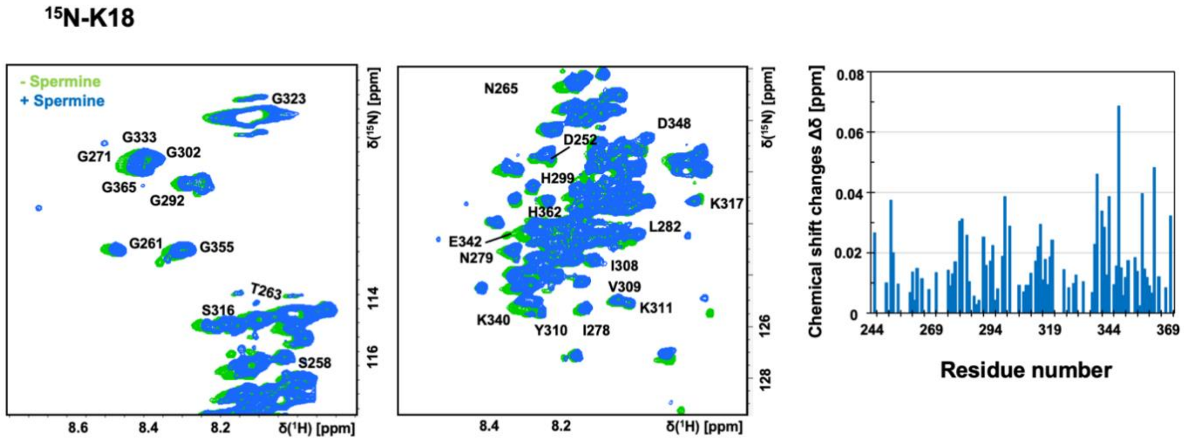

Supplementary Figure. 15: 2D ^{1}H-^{15}N-HMQC NMR measurements an interaction between spermine and K18. Spectra of ^{15}N-K18 in the absence and presence of spermine in PBS buffer with a pH 6.3. In the right panels is the chemical shift changes from the spectra presented as a function of the residue number in the protein primary sequence. The spectra were recorded at 283 K. Full spectra are shown in Supplementary Figure. 14.

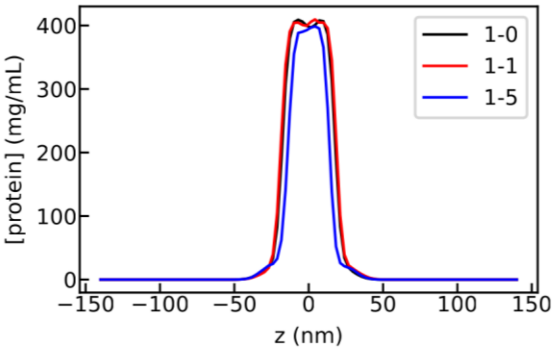

Supplementary Figure. 16: LLPS behavior of Tau at a general interaction strength of ε=0.15 kcal/mol for all the amino acids. Protein densities along z-axis of the simulation box for Tau without spermine (black line) and with spermine at 1:1 ratio (red line) and 1:5 ratio (blue line).

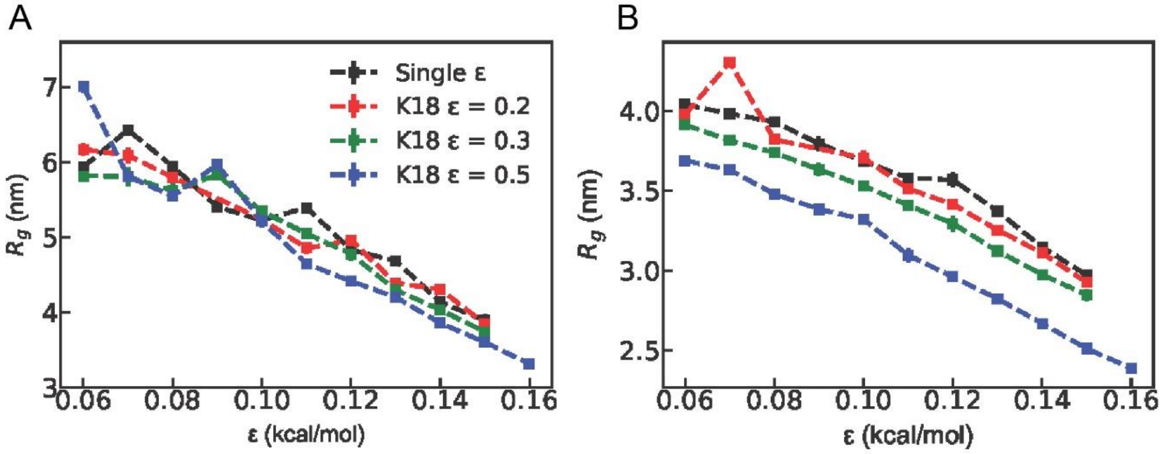

Supplementary Figure. 17. Variation of R_g when changing ε values in Tau simulations. R_g of full-length Tau are shown in (**A**) and the K18 segment in (**B**). The black dots represent results from simulations with a uniform *ε* whereas the other dots represent simulations where the *ε* for interactions between the hydrophobic residues within the K18 segment has been set to a larger value shown in the figure legend.

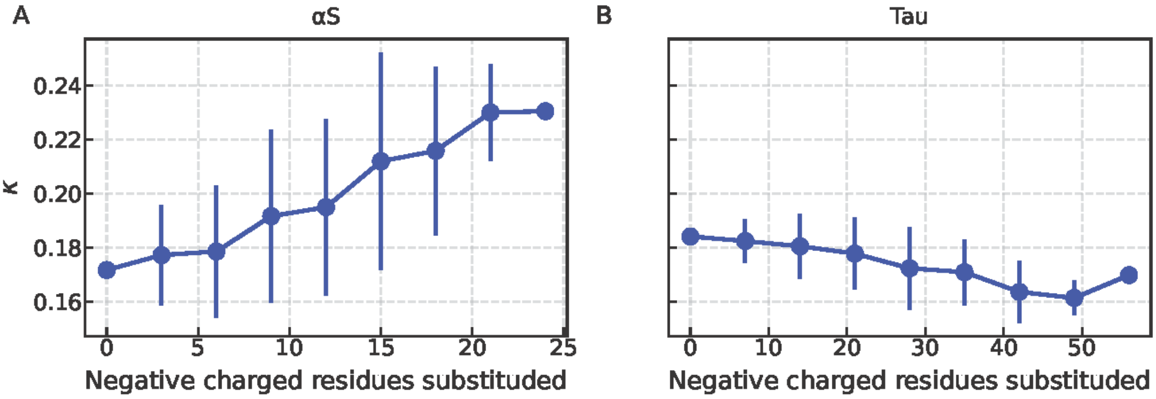

Supplementary Figure. 18: Effect of in silico neutralization of acidic residues on charge patterning (κ) for αS (A) and Tau (B). Negatively charged residues (D and E) were progressively substituted with neutral residues (N) at random positions to mimic spermine-induced charge neutralization. For each level of substitution, 100 randomized sequences were generated and *κ* values were computed. The mean and standard deviation of *κ* are shown for each substitution level.

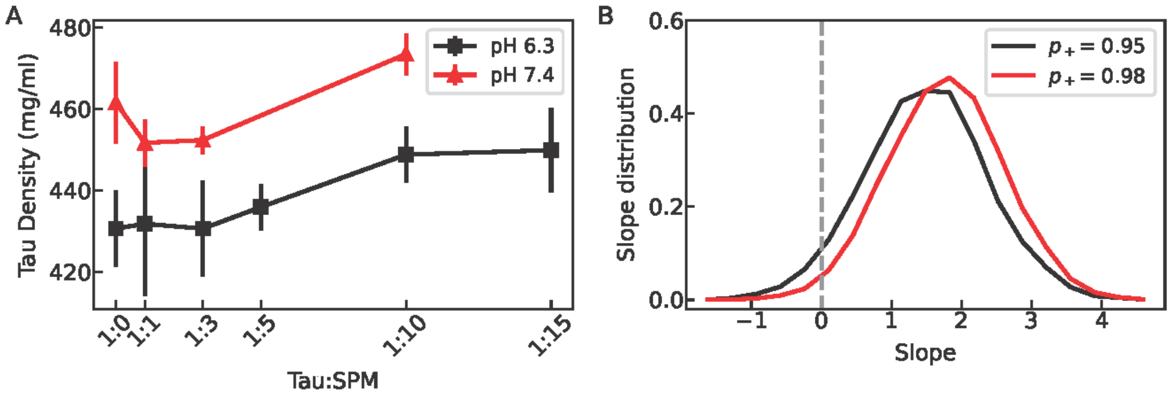

Supplementary Figure. 19: Tau phase separation at different pHs from coarse-grained simulations. (A) The densities of the Tau condensates at different pHs. (B) the histogram of linear fitting slope to the density-spermine concentration curve using a bootstrap test.

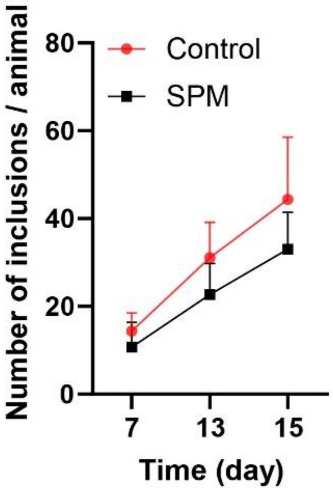

Supplementary Figure. 20: Degradation kinetics of αS condensates in *C.elegans* with and without spermine. n=15.

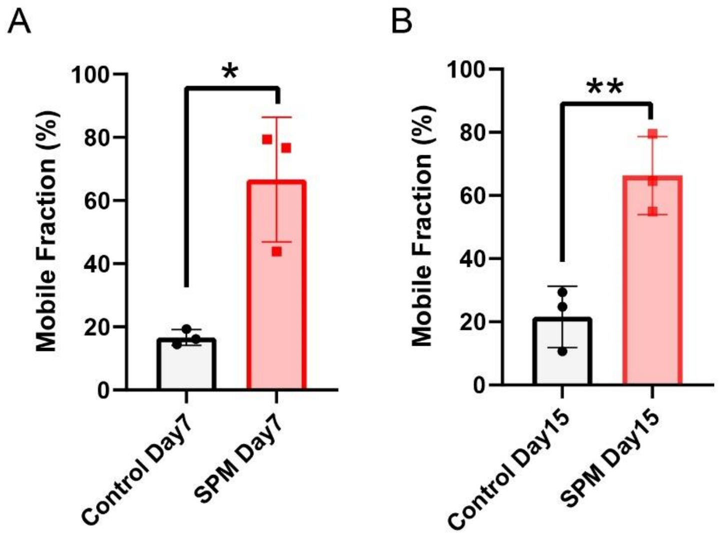

Supplementary Figure. 21: Quantification of the mobile fraction from FRAP experiments for SPM treated *C.elegans* on day 7 (A) corresponding to Figure 4D and day15 (B) corresponding to Figure 4F.

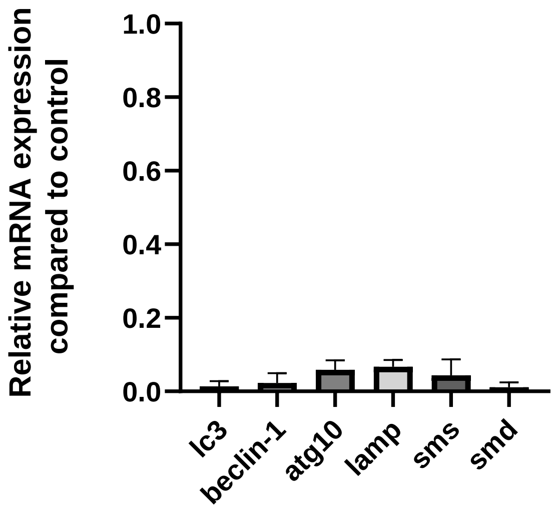

Supplementary Figure. 22: qPCR analysis confirms the efficiency of RNAi, demonstrating that gene expression levels in RNAi-treated groups were reduced to less than 10% of those observed in control groups.

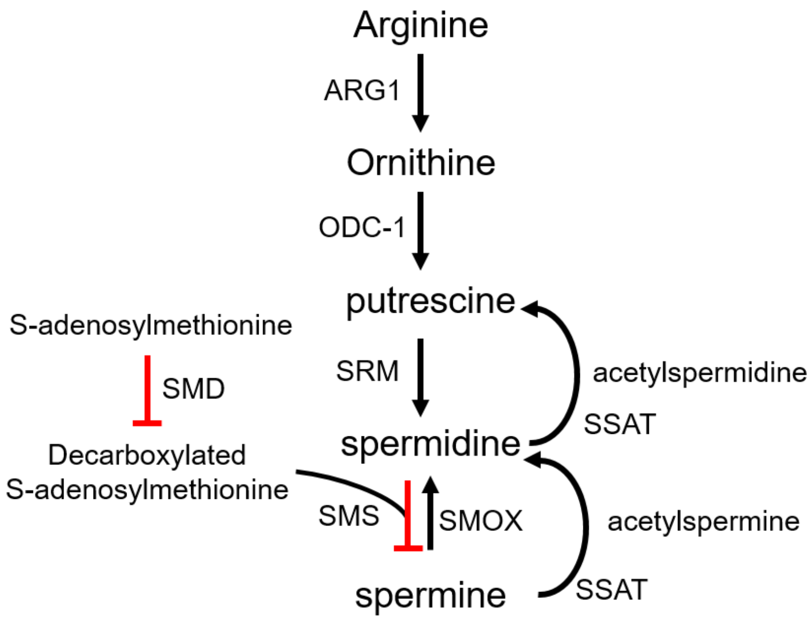

Supplementary Figure. 23: The spermine biosynthetic pathway. The *smd* and *sms* genes are outlined in red arrows, which means knock down *smd* and *sms* genes using RNAi.

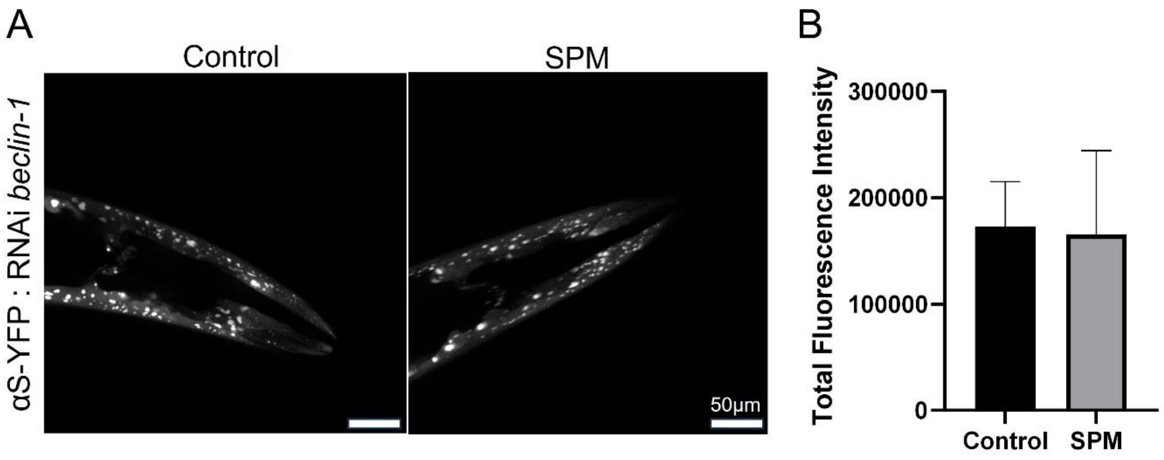

Supplementary Figure. 24: SPM treatment does not change αS-YFP condensates upon beclin-1 RNAi knockdown. A, Representative images of NL5901 *C. elegans* expressing αS-YFP in the body wall muscle, treated with beclin-1 RNAi under control or SPM-treated conditions. **B**, Quantification of total αS-YFP fluorescence intensity in control and SPM-treated animals. Data are represented as mean ± SD; no significant difference was observed between groups (ns, unpaired t-test). n=10.

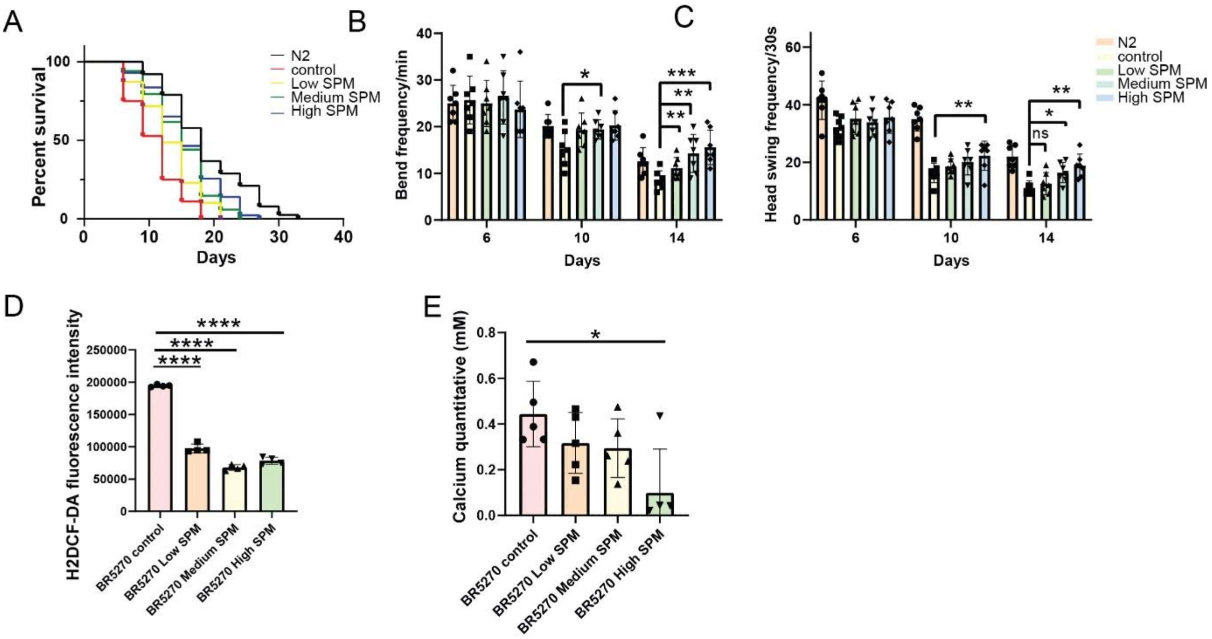

Supplementary Figure. 25: Spermine improves lifespan and rescues movement deficits and mitochondrial dysfunction in BR5270 *C.elegans* model of AD. **A**, The lifespan of Tau *C.elegans* model BR5270 treated with control (OP50 vehicle), low (100 μM), medium (200 μM) and high concentration (500 μM) of spermine, which shows that spermine improves the lifespan of Tau *C.elegans* model in a concentration dependent manner. **B-C**, Spermine rescues movement deficits in Tau *C.elegans* model. Body bend frequency (**B**), Head-swing frequency (**C**). **D-E**, Spermine rescues mitochondrial dysfunction in Tau *C.elegans* model. **D**, Reactive oxygen species (ROS), **E**, Calcium concentration.

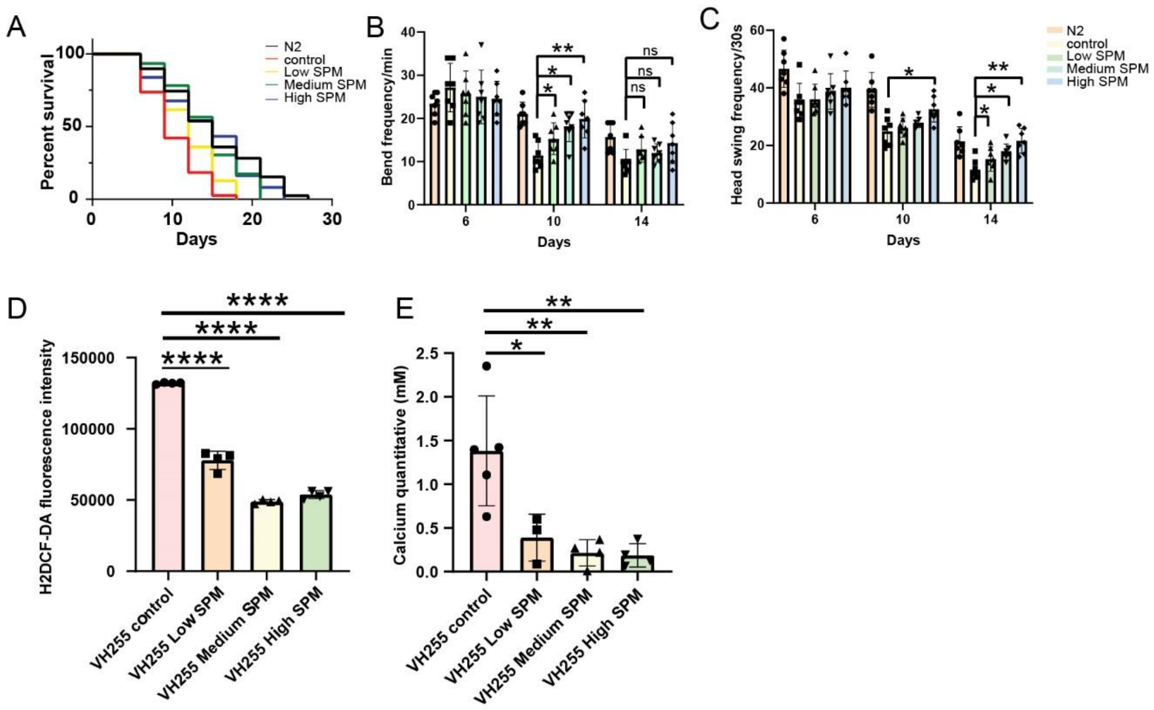

Supplementary Figure. 26: Spermine improves lifespan and rescues movement deficits and mitochondrial dysfunction in VH255 *C.elegans* model of AD. **A**, The lifespan of Tau *C.elegans* model VH255 treated with control (OP50 vehicle), low (100 μM), medium (200 μM) and high concentration (500 μM) of spermine, which shows that spermine improves the lifespan of Tau *C.elegans* model in a concentration dependent manner. **B-C**, Spermine rescues movement deficits in Tau *C.elegans* model. Body bend frequency (**B**), Head-swing frequency (**C**). **D-E**, Spermine rescues mitochondrial dysfunction in Tau *C.elegans* model. **D**, Reactive oxygen species (ROS), **E**, Calcium concentration.

Supporting Tables

Supplementary Table. 1. Summary of Multiscale Findings Across Experimental Techniques

Technique	Molecule	Experimental readout	Interpretation
NMR	Tau / αS	Residue-level chemical shift changes	SPM modulates electrostatics to favor Tau and αS LLPS
TR-SAXS	Tau / αS	*Rg* changes within 1s	SPM expands Tau and αS behavior
CG Simulation	Tau / αS	Contact map	Supports enhanced electrostatics intermolecular interaction upon SPM
LLPS Assay	Tau / αS	Phase diagram	SPM promotes Tau and αS LLPS
ThT Assay	Tau / αS	Amyloid fibril kinetics	SPM inhibits Tau and αS fibrillation
RNAi	αS	αS condensate	SPM facilitates αS condensates degradation via autophagy
FRAP	Tau / αS	Fluorescence recovery	SPM enhances Tau (in vitro) and αS (in *vitro* and *vivo*) mobility
Lifespan Assay	Tau / αS	Lifespan	SPM prolongs the lifespan of AD and PD *C.elegans* model
Behavior Assay	Tau / αS	Bend and head swing frequency	SPM improves fitness in AD and PD *C.elegans* model

Supplementary Table. 2. Primer sequences.

**Gene**	**Forward 5'-3'**	**Reverse 5'-3'**
LC3	AAACGCATCCAACTTCGTCC	CCTCGTGATGGTCCTGGTAG
LAMP	CTCTTGCTCTCGCTGCTTCT	AAACGTTGACGTCCTTGGGA
ATG10	ACGCGAGAGAGACCCATTTG	GTACGGAATGGAAGAGGGCT
Beclin1	CTGTCAGCATCCGTTGAGGT	AGAGCGTCAGAGCAATCATTACA
sms-1	AAACGCATGATGTTCCGCTG	TTTTCCGCCTTCTCCTCGTC
smd-1	CTTTGATGCGGAGGTGGAGT	TCCAAGGTGTGATCGGGTTG
Actin-1	CTA CGA ACT TCC TGA CGG ACA AG	CCG GCG GAC TCC ATA CC
